# Changes in serum inflammatory factors in acute gouty arthritis patients treated using ultrashort wave combined with loxoprofen sodium

**DOI:** 10.12669/pjms.37.7.4765

**Published:** 2021

**Authors:** Ying Wu, Yan Wang, Wenjing Yuan, Xiangzhi Xiao, Guohua Cheng

**Affiliations:** 1 Ying Wu, Clinical College, Changsha Health Vocational College, Changsha, 410600, PR China; 2 Yan Wang, Department of Integrated TCM and Western Medicine, Changsha Central Hospital, Changsha, 410014, PR China; 3 Wenjing Yuan, Department of Rehabilitation, The Fourth Hospital of Changsha, Changsha, 410006, PR China; 4 Xiangzhi Xiao, Department of Emergency, The Fourth Hospital of Changsha, Changsha, 410006, PR China; 5 Guohua Cheng, Department of Rehabilitation, The Fourth Hospital of Changsha, Changsha, 410006, PR China

**Keywords:** Gouty arthritis, Ultrashort wave, Loxoprofen Sodium, IL-1 β, IL-8, TNF-a, MMP-3

## Abstract

**Objectives:**

To study the effect of ultrashort wave combined with loxoprofen sodium on serum inflammatory factors in patients with acute gouty arthritis.

**Methods:**

Records of patients with acute gouty arthritis who were treated in The Fourth Hospital of Changsha from May 2018 to September 2020, were reviewed. Of them, 77 cases were selected and divided into two groups based on the received treatment. The control group (n=39) was treated with loxoprofen sodium, and the treatment group (n=38) was treated with an ultrashort wave combined with loxoprofen sodium, for 10 continuous days. The clinical efficacy of the treatment in two groups was analyzed.

**Results:**

After treatment, the quality of life of patients in both groups was improved (P < 0.05), but there was no significant difference in the degree of improvement between the two groups (P > 0.05). After treatment, the VAS score of the treatment group was lower than that of the control group (P < 0.05), the improvement of symptoms and signs of the treatment group was better than that of the control group (P < 0.05). Serum CRP and ESR levels in the treatment group were lower than those in the control group (P < 0.05), and the serum IL-1 β, IL-8, TNF-a and MMP-3 levels of the treatment group were lower than those of the control group (P < 0.05). The total effective rate of the treatment group (94.87%) was higher than that of the control group (87.18%), the difference was statistically significant (P < 0.05). No adverse reactions occurred in all patients during the treatment.

**Conclusion:**

An ultrashort wave combined with loxoprofen sodium in the treatment of acute gouty arthritis can reduce the inflammatory reaction, improve the degree of joint pain and swelling, improve the curative effect, and do not increase the adverse reactions. The results may be related to the regulation of IL-1 β, IL-8, TNF-a and MMP-3.

## INTRODUCTION

Gout is a type of metabolic rheumatism, a heterogeneous disease caused by the disorder of purine metabolism and / or the reduction of uric acid excretion, resulting in long-term high uric acid state and the accumulation of urate.^1^ Acute gouty arthritis (AGA) is the most severe form of this disease, with the annual incidence rate of 1.1%.^2^ Gout is more prevalent in Asian and Pacific region, with the incidence rate as high as 6% in China,^2^,^3^ while the incidence rate of gout in the United Kingdom and the United States is around 0.20%~1.70%.^3^

In vivo studies show that gout results from monosodium urate (MSU) crystals accumulating excessively in joint capsule, synovial capsule, cartilage and other tissues, resulting in lesions and inflammatory reactions of soft tissues around the joint.^4^ There is strong evidence that inflammatory cytokines, such as IL-1 β, IL-8, TNF-a and MMP3 play an important role in the gout pathogenesis.^5^ The release of inflammatory cytokines that triggers rapid recruitment of neutrophils to the site of MSU deposition, is the core mechanism of gouty arthritis.^6^ Most of the studies of AGA therapy efficiency take symptom score and total effective rate as outcome indicators. While nonsteroidal anti-inflammatory drugs, such as loxoprofen sodium, are considered a first-line gout treatment options that aim at reducing inflammation as quickly as possible,^5^ they are associated with risks of gastrointestinal and cardiovascular side effects.^7^

Recent clinical reports have proved that the application of physical factors (light, electricity, heat, etc.) in the treatment of AGA has achieved good results with less toxicity and side effects.^8^ Among these methods, ultrashortwave therapy is one of the most widely used high frequency electrotherapies. A combination of oral medication and ultrashort wave therapy resulted in significantly improved symptoms and joint function of patients with AGA as compared to simple drug therapy alone. Moreover, combination therapy successfully reduced the levels of C-reactive protein, serum uric acid and erythrocyte sedimentation rate in AGA patients.^9^ The main goal of this study was to retrospectively evaluate the efficacy of loxoprofen sodium in combination with ultrashort wave therapy for treatment of AGA using inflammatory cytokines, closely related to gout acute inflammation, as the efficacy evaluation indicators.

## METHODS

This retrospective study analyzed records of the patients that were diagnosed with gouty arthritis (acute phase) in the Changsha Fourth Hospital from May 2018 to September 2020. Of all cases, 38 patients whose records indicated treatment with loxoprofen sodium tablets 60mg, 3 / D orally. were retrospectively selected as the control group. Thirty nine patients that were treated with loxoprofen sodium tablets 60mg, 3 / D orally in combination with the ultrashort wave [(cd-1a-a ultrashort wave therapeutic apparatus by Changchun Aier Medical Appliance Co., Ltd.), with the inductive electrode placed opposite to the diseased joint with the gap of 2-3cm, no heat, 15min / day], were selected as the treatment group. Patients in both groups were treated for 10 days. The study was approved by the hospital ethics committee (Approval number CSSDSYY-LLSC-KYXM-2020-02-02, Changsha Fourth Hospital).

### Diagnostic criteria

All the patients were assessed using 2015 Gout Classification Criteria, with a maximum score of 23 points, including 16 points for clinical symptoms, 9 points for laboratory examination and 8 points for imaging performance. A threshold score of ≥ 8 classifies an individual as having gout.

### Inclusion criteria

(1) Patients diagnosed with gout, (2) age 18-65 years, (3) patients with acute attack within 48 hours without any other special treatment.

### Exclusion criteria

(1) Pregnant and lactating women; (2) patients allergic to the drug; (3) gout secondary to leukemia, myeloma, hemolytic anemia, cancer, or chemotherapy- and radiotherapy-caused; (4) peptic ulcer in active phase; (5) gout diagnosis combined with cardiovascular, cerebrovascular, liver, kidney, hematopoietic system and other serious conditions and mental health disorder; (6) participants in other clinical drug research studies; (7) patients with poor compliance; (8) serious adverse events and complications occurred during the treatment process; or patients deemed not suitable for further treatment due to special physiological changes; (9) patients lost to follow-up.

### Patient Evaluation and Management

Basic management of all patients in the study included the following general advises: bed rest, physical activity restriction, no overeating, alcohol consumption and stress, comfortable footwear, no aspirin, triazolam diuretics and other drugs that may affect uric acid excretion; no seafood, thick broths, animal viscera, clams, oyster, all kinds of bean products and strong tea; low calorie diet, limiting the intake of fat and protein, especially animal protein; proper hydration, alkaline drinks or oral sodium bicarbonate tablets to promote uric acid excretion. Patients were encouraged to actively treat diabetes, hypertension, coronary heart disease and other accompanying diseases.

### Clinical evaluation

For all the patients, records indicated that the clinical evaluation of pain symptoms and joint function was performed before the beginning of treatment and after 10 days of treatment. For assessment of pain, the following scales were used: SF-36 Health Survey short form, with higher score indicating better corresponding field status; Vas visual analogue scale: 0 points for completely painless, 10 points for intolerable severe pain. Main symptoms and signs of joint swelling were also scored. The score of joint tenderness was as follows: 0 points: no tenderness, no pain during heavy pressure or maximum passive activity; one points: mild tenderness; two points: moderate tenderness, patients with heavy pressure complained of pain with frown and other discomfort expression; three points: no tenderness, no pain during heavy pressure or maximum passive activity three points: mild tenderness; three points: moderate tenderness, patients with heavy pressure complained of pain with frown and other discomfort expression; three points: severe tenderness and avoidance or refusal of examination. The involved joints were evaluated with the above scoring criteria and the average score was taken. Joint swelling score: 0: no swelling or disappearance of swelling; 1: slight swelling of joint, shallow skin texture, bone markers of joint are still obvious; 2: moderate swelling of joint, clear swelling of joint, skin texture basically disappeared, bone markers are not obvious; 3: Severe swelling of joints, tight skin and disappearance of bone markers. The swelling joints were evaluated before and after treatment with the above scoring criteria and the average score was taken.

### Evaluation of laboratory indicators

On the day before the treatment and the second day after the end of the whole course of treatment, fasting blood samples were collected from the median cubital vein of the patients to detect the levels of CRP, ESR, IL-1 β, IL-8, TNF-a and MMP-3. TNF-a and MMP-3 were used as evaluation indexes. IL-1 β, IL-8, TNF-a and MMP-3 levels were detected by commercially available multiplex electrochemiluminescence immunoassays (Meso Scale Discovery [MSD], Rockville, MD, USA) using iMark absorption light microplate reader (Bio Rad, Hercules, CA, US). The assay was performed according to the manufacturer’s recommendations.

### Total efficacy evaluation

After calculating the average percentage of improvement of the above indicators, the efficacy evaluation was divided into the following four grades: a. markedly effective: the improvement rate of indicators was more than 75%; b. effective: the improvement rate of indicators was 50% - 75% (including 50%); c. improved: the improvement rate of indicators was 30% - 50% (including 30%); d. ineffective: the improvement rate of indicators was less than 30%. The following equation was used:

Index improvement rate = (before treatment - after treatment) / before treatment X 100%; B. total effective rate = (improved + effective + markedly effective) / total cases x 100%.

### Statistical analysis

SPSS 21.0 statistical software was used for data analysis. Measurement data were expressed as mean ± standard deviation (x ± s). Two independent samples t test was used for comparison between groups, paired t test was used for comparison before and after treatment in groups, and rank sum test was used if it was not normal. Chi square test or rank sum test were used for count data; P < 0.05 showed that the difference was statistically significant.

## RESULTS

A total of 77 subjects were included in this study, 38 in the control group and 39 in the treatment group. There were no adverse reactions reported in both groups. There was no significant difference in gender and age between the two groups (P > 0.05). The treatment group contained 37 males and 2 females, while the control group contained 37 males and 1 female. The average age of patients in the treatment and the control group was N(SD) 47.13 (10.02) and 46.25 (11.21) respectively.

### Pain, Symptoms, and Quality of Life

Quality of life was assessed using the SF-36 health survey. Parameters assessed were physical health (PF), physical role function (RP), physical health (PF), physical role function (RP), Physical pain (BP), general health (GH), vitality (VT), social function (SF), emotional role function (RE) and mental health (MH). Eight dimensions were used to evaluate the quality of life of patients before and after treatment. As shown in Table-I, paired sample t-test showed a statistically significant improvement in the quality of life scores of both groups (P < 0.05). However, there was no significant difference in the degree of improvement between the two groups by independent sample t-test (P > 0.05).

**Table I:** Comparison of quality of life between the two groups (x ¯ ± s, points).

*Quality of life factor*	*Treatment group*		*Control group*		*t/p Comparison of treatment group before and after treatment*	*t/p Comparison of control group before and after treatment*	*t/p Comparison of treatment group and control group after treatment*

	*Before treatment*	*After treatment*	*Before treatment*	*After treatment*			
Physiological function (PF)	37.96±20.11	68.21±30.11	37.88±19.86	69.54±29.88	5.217/0.000	5.440/0.000	0.195/0.000
Physiological function (RP)	30.11±24.23	60.22±37.23	30.18±23.99	60.35±36.98	4.233/0.000	4.219/0.000	0.015/0.988
Somatic pain (BP)	28.78±18.11	60.11±29.15	29.01±17.89	61.06±28.17	5.701/0.000	5.920/0.000	0.145/0.885
General health (GH)	30.58±23.45	47.25±33.58	30.37±22.98	46.95±31.42	2.542/0.013	2.626/0.011	0.040/0.968
Livepower (VT)	25.68±24.89	52.77±34.87	26.47±25.12	53.36±35.14	3.875/0.000	3.837/0.000	0.073/0.942
Social function (SF)	30.87±26.47	64.25±38.74	30.15±26.65	63.84±37.22	4.443/0.000	4.537/0.000	0.047/0.962
Emotional intelligence (RE)	31.01±21.11	44.25±31.65	30.56±20.94	43.65±32.06	2.173/0.033	2.224/0.029	0.083/0.934
Mental health (MH)	32.45±21.03	52.66±23.65	31.91±21.01	53.45±22.98	3.988/0.000	4.264/0.000	0.149/0.882

Pre-treatment VAS score of both groups (7.33±1.33 for treatment and 7.24±1.15 for control) was similar as indicated by the independent sample t test (P > 0.05). After the treatment, the paired sample test showed a significant decrease in the VAS score in both groups (2.34±0.42 for treatment and 2.85±0.62 for control, P < 0.05), indicating that the VAS scores of the two groups were improved after treatment. Independent sample test indicated that the post-treatment VAS score of the treatment group was significantly lower than that of the control group (P < 0.05). As shown in Table-II, there was no significant difference in the scores of symptoms and signs between the two groups before treatment (P > 0.05). After treatment, the joint tenderness score and joint swelling score of the two groups were significantly lower than before treatment, and the difference was statistically significant in the same group before and after treatment (P < 0.05). After the treatment, the reported improvement in symptoms and signs in the treatment group was markedly better than that in the control group (P < 0.05), Table-II.

**Table II:** Comparison of improvement of main symptoms and signs between the two groups (x ± s, points).

*Main symptoms*	*Treatment group*		*Control group*		*t/p Comparison of treatment group before and after treatment*	*t/p Comparison of control group before and after treatment*	*t/p Comparison of treatment group and control group after treatment*

	*Before treatment*	*After treatment*	*Before treatment*	*After treatment*			
Joint tenderness	1.77±0.71	0.61±0.51	1.65±0.75	0.60±0.53	8.287/0.000	7.048/0.000	0.084/0.933
Joint swelling	1.93±0.67	1.11±0.65	1.87±0.72	1.21±0.65	5.486/0.000	4.194/0.000	0.675/0.502

### Serum Analysis

There was no significant difference in ESR and CRP levels between the two groups before treatment (P > 0.05). After the treatment, the levels of ESR and CRP in the two groups were significantly lower than before treatment, and the difference was statistically significant in the same group (P < 0.05); after treatment, the levels of CRP and ESR in the treatment group were lower than those in the control group (P < 0.05), indicating that the treatment group was better than the control group in reducing CRP and ESR (Table-III) As shown in Table-IV, there was no significant difference in the levels of IL-1 β, IL-8, TNF-a and MMP-3 between the two groups before treatment (P > 0.05). After the treatment, serum levels of inflammatory factors in the two groups were significantly lower than before treatment, and the difference was statistically significant in the same group (P < 0.05); after the treatment, the levels of inflammatory factors in the treatment group were lower than those in the control group, P < 0.05, indicating that the treatment group was better than the control group in reducing inflammatory factors such as IL-1 β, IL-8, TNF-a and MMP-3, Table-IV.

**Table III:** Comparison of ESR and CRP levels between the two groups (x ± s).

*Group*	*CRP(mg/L)*		*t*	*p*	*ESR(mm/h)*		*t*	*p*
	
	*Before treatment*	*After treatment*			*Before treatment*	*After treatment*		
Treatment group	38.26±8.77	8.25±4.22	19.256	<0.001	51.41±16.32	25.36±8.85	8.763	<0.001
Control group	36.95±7.56	11.66±5.33	16.854	<0.001	52.65±18.32	32.65±10.21	5.878	<0.001
t	0.702	3.117	-	-	0.314	3.351	-	-
p	0.485	0.003	-	-	0.755	0.001	-	-

**Table IV:** Comparison of serum inflammatory factors between the two groups (x ± s).

*Group*		*IL-1β (pg/mL)*	*IL-8 (pg/mL)*	*TNF-a (ng/mL)*	*MMP-3 (ng/mL)*
Treatment group	Before treatment	61.52±8.37	49.25±7.01	346.9±21.66	124.63±15.22
	After treatment	31.56±6.11	28.35±5.11	212.3±34.68	62.20±13.15
Control group	Before treatment	62.33±8.14	46.12±7.22	356.1±22.53	118.95±19.16
	After treatment	40.49±7.15	34.98±6.45	253.5±25.40	80.23±20.13
t/p comparison of treatment group before and after treatment		18.055/0.000	15.046/0.000	20.558/0.000	20.004/0.000
t/p comparison of control group before and after treatment		12.426/0.000	7.093/0.000	18.628/0.000	8.589/0.000
t/p comparison of treatment group and control group after treatment		5.897/0.000	5.006/0.000	5.935/0.000	5.182/0.000

### Overall Treatment Efficacy

Of 39 cases in the treatment group, there were 29 remarkable effect cases, 8 effective cases, and 2 invalid cases. This contrasted with 20 remarkable effect cases, 14 effective cases, and 4 invalid cases out of a total of 38 control group cases. The total effective rate of the treatment group (94.87%) was slightly higher than that of the control group (89.47%), but the difference was not statistically significant (P>0.05).

## DISCUSSION

This study showed that ultrashort wave therapy combined with NSAIDs for treating AGA can significantly improve symptoms and contribute to the recovery of joint function compared to NSAID treatment alone. The study therefore highlights a new and potentially better therapeutic approach for treating AGA.

Gout is a chronic disease of monosodium urate (MSU) crystals deposition. MSU crystals, produced as a result of purine metabolic disbalance, stimulate influx of monocyte macrophages and neutrophils into the diseased joint, leading to secretion of inflammatory factors such as IL-1 β, IL-8, TNF - α and MMP3. IL-1 β plays an important role in the process of neutrophil chemotaxis and activation.^10^ IL-1 β is an important mediator that regulates the initiation of inflammation and inflammatory response, and plays an important role in both acute and chronic gouty arthritis. It not only starts the inflammatory response, but also aggravates the inflammatory response in vivo by stimulating the release of other proinflammatory mediators, inducing the degradation of cartilage extracellular matrix.^11^,^12^ IL-8 is a chemokine that can activate neutrophils and eosinophils and participate in inflammatory response.^12^ TNF-α is an inflammatory mediator produced by a variety of immune cells that participates in a variety of inflammatory reactions, including the occurrence and development of AGA inflammatory response.^13^,^14^ MMP-3 belongs to the common type of human serum proteolytic enzymes that can degrade the extracellular matrix in the lesion site, causing the destruction of articular cartilage. The increase in the MMP-3 levels can timely and accurately reflect the degree of inflammatory reaction of synovium in the lesion site.^15^,^16^ In conclusion, the study shows that serum inflammatory mediators such as IL-1 β, IL-8, TNF - α and MMP-3 play an important role in the pathological process of AGA and can reflect the clinical efficacy. Therefore, we believe that the expression levels of IL-1 β, IL-8, TNF - α and MMP-3 in peripheral serum may be objective indicators of clinical efficacy of AGA.

With the continuous development of rehabilitation medicine, physical therapies in general, and ultrashort wave therapy in particular play an increasingly important role in the treatment of gouty arthritis with good results. Ultrashort wave therapy uses specific wavelength and frequency of electromagnetic waves for treatment.^17^,^18^ The physiological effects of this therapy include dilating blood vessels, increasing vascular wall permeability, improving blood circulation, eliminating inflammation, stimulating metabolism, increasing connective tissue compliance and joint range of motion, reducing joint stiffness, inhibiting sensory nerves and blocking pain impulse conduction to relieve pain.^19^ The general belief is that the effect of pulse type ultrashort waves on the human body is mainly based on the concussion effect of the pulse group, that leads to enhanced cell activity without heat sensation during treatment.^20^ This non thermal effect of non-caloric ultrashort wave can not only enhance the activity of phagocytes, but also enhance the activity of alkaline phosphatase in peripheral blood leukocytes, thus promoting the absorption and dissipation of local inflammation and accelerating the repair of damaged tissues.^18^ In the recent study we found that ultrashort wave treatment of AGA can significantly reduce the symptoms of redness, swelling, heat and pain, shorten the course of disease, and is easy to operate, non-invasive and painless, which makes this therapy easily acceptable by most of the patients. However, more in-depth larger-scale clinical studies are needed to fully evaluate the potential of ultrashort wave therapy on AGA.

### Limitation of the study

The main limitation of the current study is its retrospective nature with a relatively small sample size of only 77 individuals. Small sample size could also explain insignificant difference in the total clinical effect between two groups. Longer follow-up multi-center studies are required to investigate long-term patient health as well as potential relapse incidence.

## CONCLUSION

Our study shows that, compared with the simple use of NSAIDs, ultrashort wave therapy combined with NSAIDs in the treatment of acute gouty arthritis can significantly improve the symptoms and signs of gout patients, and contribute to the recovery of joint function. The most significant effect was observed on the decrease of CRP and ESR. The clinical efficacy of ultrashort wave therapy may be closely related to the reduction of IL-1 β, IL-8, TNF - α, MMP-3 levels and the inhibition of local inflammatory reaction. This study may further contribute to providing some basis and reference for the future clinical treatments using ultrashort wave therapy and to the basic research of ultrashort wave biomolecules.

### Authors’ Contributions:

**YW:** Designed the project.

**YW,** **WY, & XX:** Were involved in data collection and data analysis.

**YW:** Prepared the manuscript.

**GC:** Edited the manuscript; all authors read and approved the final manuscript.

**GC:** Is responsible and accountable for the accuracy or integrity of the work.
